# Perioperative use of steroids in neonatal heart surgery: Evidence based practice or tradition?

**DOI:** 10.1016/j.amsu.2016.07.003

**Published:** 2016-07-06

**Authors:** Daniel Fudulu, Alvin Schadenberg, Gianni Angelini, Serban Stoica

**Affiliations:** a Bristol Heart Institute, Bristol, UK; b Bristol Royal Hospital for Children, Department of Paediatric Intensive Care, Bristol, UK; c Bristol Royal Hospital for Children, Department of Paediatric Cardiothoracic Surgery, Bristol, UK

**Keywords:** Review, Steroids, Neonates, Heart surgery, Cardiopulmonary by-pass outcomes, Evidence

## Abstract

A best evidence topic was written according to a structured protocol. The question addressed was: Is the use of prophylactic, perioperative steroids associated with better clinical outcomes following heart surgery in neonates? Altogether, 194 papers were found using the reported search, of which 8 represented the best evidence to answer the clinical question. One study found improved hospital survival in the group without steroids. Steroids increased infection in one large retrospective study. Incidence of hyperglycaemia was increased in the steroid group in 2 out of 5 studies. Use of steroids was associated with a shorter duration of ventilation and better oxygenation in one study. Postoperative steroid infusion was associated with reduced low cardiac output syndrome, inotrope requirement and less fluid retention in two controlled trials in which all patients received preoperative steroid. High dose steroid was associated with renal dysfunction in one study, comparing single versus double dose steroid prophylaxis. Steroid non-recipients had a shorter intensive care length of stay in 2 out of 7 studies. We conclude that use of steroids perioperatively does not unequivocally improve clinical outcome in neonatal heart surgery. A large, multicentre prospective randomized controlled trial is needed to clarify the role of steroids in paediatric heart surgery.

## Highlights

• Prophylactic glucocorticoids are widely used to modulate the inflammatory response and treat presumed relative adrenal insufficiency in children undergoing heart surgery.
• There is wide variability between centre and physician's preference in the timing, dose, route or type of steroid to be administered.
• There is an intense debate about prophylactic steroid use and their effect on the clinical outcomes.
• The weight of the current evidence for prophylactic, perioperative steroid administration suggests they do not unequivocally improve clinical endpoints.
• A large, multicentre prospective randomized controlled trial is needed to clarify the role of steroids in paediatric heart surgery.

## 1 Introduction

A best evidence topic was constructed according to a structured protocol. This is fully described in a previous publication in the IJS([1]).

## 2 Three-part question

In neonates undergoing heart surgery with cardiopulmonary by-pass (CPB) is prophylactic use of perioperative steroids associated with better clinical outcomes?

## 3 Clinical scenario

You are the registrar scrubbing for an arterial switch operation in a 14 days old neonate. You notice the anaesthetist administering dexamethasone before induction. The consultant explains that he gives steroids in all neonates to suppress systemic inflammatory response and prevent effects of potential adrenal insufficiency. However, you scrubbed in similar cases and other anaesthetists do not give steroids at all. You ask yourself if the use of perioperative steroids improves clinical outcomes following heart surgery in neonates? You resolve to check the literature yourself.

## 4 Search strategy

Medline database using PubMed interface, search field: title/abstract.

“(steroid OR corticosteroid OR methylprednisolone OR dexamethasone OR hydrocortisone) AND (neonate OR neonatal OR children OR pediatric) AND (by-pass OR cardiopulmonary bypass OR cardiac surgery OR cardiac surgical procedure OR heart surgery OR congenital heart disease OR pediatric heart surgery)”.

## 5 Search outcome

We found 194 papers using the reported search. From these, 8 papers were identified that provided the best evidence to answer the question. The main outcome of these papers are presented in Table 1.

## 6 Results

This evidence review focuses on prophylactic, perioperative steroid regimes for neonatal cardiac surgery. The abrupt interruption of maternal corticotropin releasing factor and cortisol at parturition and the immaturity of the hypothalamic-pituitary axis have been related to the reduced ability of neonates to mount an adequate stress response [2]. There is no consensus in diagnosing adrenocortical insufficiency in children and correlation with clinical outcome is controversial [2]. Cardiac surgery with use of cardiopulmonary bypass (CPB) provokes a systemic inflammatory response. Steroids have been widely used to mitigate potential deleterious effects of systemic inflammation [3], [4]. However, the impact of steroids on clinical outcomes following neonatal heart surgery remains unclear.

We found only two randomized controlled trials assessing the effect of steroids versus no steroids on clinical outcomes [5], [6] in neonates. These trials had a small sample size and were single centre. The majority of the studies were retrospective, three of them being multicentre on large cohorts [3], [4], [7]. There was heterogeneity among the studies in the clinical outcomes measured. Most studies measured: early mortality, ventilator duration, infection rates, hyperglycaemia and insulin requirement, low cardiac output prevalence, fluid balance and intensive care unit (ICU) length of stay. Furthermore, we found variability in the type, dose and route of steroid regimen. This is similar to previous surveys of steroid use [8], [9].

Keski-Nisula et al. [6], randomized 40 neonates to either receiving methylprednisolone at induction or placebo. In the steroid group, blood glucose levels were significantly higher compared to placebo. There was no difference in early mortality, inotropic score, serum lactate, duration of ventilation or ICU stay.

Graham et al. [10], randomized neonates to receive methylprednisolone as either 2-dose (n = 39), being 8 h preoperatively and included to the CPB prime, or single dose (n = 37) being included in the CPB prime only. The 2-dose steroid cohort had significantly higher serum creatinine and reduced diuresis. Mortality, infection rates, insulin requirements, inotropic requirement, fluid balance, duration of mechanical ventilation, ICU and hospital stay did not differ between the groups.

Robert et al. [5], randomized 40 neonates undergoing cardiac surgery with CPB to either postoperative hydrocortisone infusion for 5 days (n = 19) or placebo (n = 21). The hydrocortisone group had improved fluid balance, urine output and inotropic score. No significant differences were found in: mortality, ventilator-free days, hospital length of stay, kidney function, antibiotic use for suspected infections or blood glucose levels.

Pasquali et al. [3], in a multivariate analysis of the largest population to date: 46730 children (10018 neonates) undergoing cardiac surgery, compared outcomes between steroid recipients and non-recipients. In the neonatal analysis, steroids increased ICU stay and the use of insulin, however there were no differences in infection rates, duration of ventilation or early mortality.

Pasquali et al. [4] analysed steroid use in 3180 neonates undergoing heart surgery. There was no significant mortality or length of stay difference between any methylprednisolone regimens versus no steroids. The authors stratified the neonates using a risk score and performed subgroup analyses. The lower surgical risk group had a significant association with infection across all steroid regimens.

Elhoff et al. [7], using data from a trial database, analysed outcomes following the Norwood procedure in neonates that received intraoperative steroids (n = 498) compared to non-recipients (n = 51). In the univariate analysis non steroid recipients had better survival but longer ICU and hospital stays. In multivariate analysis, hospital survival again trended toward favouring the non-steroid group, while length of stays, ECMO, infection and renal failure rates no longer differed statistically.

Dreher et al. [11] compared outcomes from neonates undergoing heart surgery with (n = 55) and without (n = 58) methylprednisolone added to the CPB prime. There were no differences in clinical outcomes (including length of stay, ventilation requirement, infection and mortality) between both groups.

Ando et al. [12] enrolled 20 neonates undergoing biventricular repair. Ten neonates were assigned to receive either hydrocortisone infusion after bypass or placebo infusion. The placebo group had a reduction in the left ventricular shortening fraction, had higher inotropic requirements and lactate levels compared to the steroid groups. Furthermore, steroid supplementation was associated with less body oedema, higher blood oxygenation and shorter duration of ventilation. There was no difference in renal failure or blood glucose levels.

## 7 Clinical bottom line

The majority of the studies suggest that perioperative steroid use does not affect early mortality in neonates undergoing heart surgery. One large retrospective study highlighted increased infection in steroid recipients. A few small studies suggested perioperative steroids protected against low cardiac output syndrome and reduced fluid retention. One small study demonstrated reduced ventilation times and better oxygenation in the steroid group while the rest of the papers showed no difference. In two studies, steroid use increased ICU stay. One study suggested worsening of the renal function in the high dose steroid group. Given the weight of current evidence, prophylactic, perioperative steroid administration for neonatal heart surgery does not unequivocally improve clinical endpoints. A large, multicentre prospective randomized controlled trial is needed to clarify the role of steroids in paediatric heart surgery.

## Funding

The study was supported by the British Heart Foundation and the NIHR Bristol Cardiovascular Biomedical Research Unit.

## Figures and Tables

**Table 1:** Best evidence papers.

Author, date and country	Patient group	Study type and level of evidence	Outcomes	Key results	Comments
Keski-Nisula et al., June 2013, Ann Thorac Surg, Finland (6)	40 neonates randomized into 2 groups: 1 group received intravenous methylprednisolone (30 mg/kg of methylprednisolone) and the other a placebo.	Double blinded PRCT (Level 2)	Blood glucose levels lactate, inotropic score, lactate, duration of mechanical ventilation, length of ICU stay, mortality at 30 days.	Blood glucose levels: methylprednisolone (MP) group (11.6 ± 3.0) vs placebo group (8.7 ± 2.9), p = 00.5. MP group vs. Placebo: early mortality (0 vs 3, p = 0.231), inotrope score (15.2 ± 8.2 vs. 16.5 ± 9.6, p = 0.645), lactate levels (2.7 ± 1.0 vs. 2.1 ± 0.8,p = 0.069), duration of ventilation (5.6 4.2 vs. 5.7 ± 4.6, p = 0.998) or length of ICU stay (9.3 ± 5.2 vs. 8.2 ± 4.9, p = 0.515).	No fluid balance or renal function outcomes.
Graham et al., May 2011, J Thorac Cardiovasc Surg, USA (10)	76 neonates assigned to receive either two-dose (8 h preoperatively and operatively, n = 39) or single dose (operatively = 37) of methylprednisolone (30 mg/kg per dose)	Double blinded PRCT (Level 2)	Low cardiac output, serum creatinine, postoperative diuresis, infection, insulin requirements, postoperative diuresis, death at 30 days, inotropic score, fluid balance, duration of mechanical ventilation, intensive care unit and hospital stay.	Single vs two dose MP: serum creatinine (0.53 ± 0.12 mg/dl, p = 0.03 vs. 0.61 ± 0.18 mg/dl p = 0.03, between-group differential urine output (−96 ± 49 ml, p = 0.05), low cardiac output syndrome incidence (46% (17/37) vs. 38% (15/39), p = 0.51), infection rates: 5 (14%) vs. 5 (13%), p = 0.96; insulin drip 0 vs. 3 (8%), p = 0.24; highest lactate (mmol/L): 3.8 ± 2.4 vs 5.2 ± 3.5, p = 0.05; total fluid in at 36 h (mL) 575 ± 145 vs. 586 ± 156, p = 0.77; total fluid out at 36 h (mL): 600 ± 250 vs. 558 ± 203, p = 0.43; duration of mechanical ventilation: 5.8 ± 7.9 6.8 ± 9.5, p = 0 0.21; intensive care unit stay: 11.0 ± 18.5 vs. 10.8 ± 12.8, p = 0.19, hospital stay: 23 ± 25 vs. 22 ± 15 0.34, p = 0.34.	Single versus double dose steroid study design.
Robert et al., Sept 2015, Pediatr. Crit Care Med, USA (5)	40 neonates were randomized: 19 to hydrocortisone infusion or 21 to placebo infusion. The steroid group had a hydrocortisone bolus (50 mg/m^2^) after weaning from CPB followed by a 48 h hydrocortisone infusion tapered over 3 days (40 mg/m^2^/d × 24 h,30 mg/m^2^/d × 12 h, 20 mg/m^2^/d × 12 h,10 mg/m^2^/d × 24 h, then stop)	Double-blinded PRCT (Level 2)	Low cardiac output, fluid balance, urine output syndrome, inotropic score, mortality, duration of ventilation, length of stay on intensive care unit, renal failure, antibiotics use for infection, blood glucose.	Hydrocortisone vs. placebo group: low cardiac output syndrome (5/19 (26%) vs. 12/21 (57%), p = 0.049), negative net fluid balance at 48 h (−114 vs −64 ml/kg; p = 0.01), urine output at 0–24 h (2.7 vs 1.2 ml/kg/hr; p = 0.03), mortality (0 vs. 3, p = 0.23), time until first extubation: 51 (interquartile range (IQR), 34–83) vs. 55 (IQR, 21–195), p = 0.7; hospital length of stay: 19 (IQR, 9–24) vs. 13.5 (IQR, 9–24); acute kidney injury: 7 (37%) vs. 7 (33%), p = 1, antibiotics for suspected infection: 7 (37%) vs. 8 (38%), p = 1; blood glucose levels: 10 (53%) vs. 15 (71%), p = 0.3.	Both arms received methylprednisolone (10 mg/kg) 8 h and 1 h prior to their operations. No patient received intraoperative steroids. The hydrocortisone group was weaned off vasopressors sooner with a difference in inotrope-free subjects seen after 48 h (p = 0.033).
Pasquali et al., Circulation, November 2010, USA (3)	46730 children (10018 neonates) - outcomes between steroids recipients (54%) and non-recipients were compared. Multivariate analysis, adjusted for propensity score and individual covariates was used. Patients were stratified using the Risk Adjustment in Congenital Heart Score.	Observational study (Level 3)	ICU length of stay, use of insulin, In-hospital mortality, duration of ventilation, postoperative length of stay and infection.	Least square means difference: steroids vs no steroid (adjusted outcomes): ICU length of stay = 2.5 (1.34–3.66, p < 0.001); postoperative insulin: 2.32 (1.97–2.73), p < 0.001); duration of ventilation = 1.11 (−2.47–0.25),p = 0.11; infection = 0.85 (0.68–1.05, p = 0.14 Mortality, odds ratio (95% confidence interval): 1.07 (0.89–1.27), p = 0.48.	Steroid dose and regimens not reported. No outcomes on inotrope requirements, low cardiac output syndrome, fluid retention or renal failure.
Pasquali et al., Pediatrics. February 2012, 2012, USA (4)	3180 neonates: 22% received methylprednisolone on both the day before and day of surgery, 12% on the day before surgery only, and 28% on the day of surgery only; 38% did not receive any steroids.	Multicentre retrospective study (Level 3)	In-hospital mortality, total length of stay, ICU length of stay, infection.	Adjusted postoperative outcomes meyhylprednisolone receipients compared to non-steroid recipients: In hospital mortality, (adjusted odds ratio 95% CI): both day of/before surgery 1.00 (0.66–1.50), p = 0.99, day before surgery 0.95 (0.60–1.52), p = 0.84, day of surgery 1.28 (0.93–1.75), p = 0.13. Total length of stay, (adjusted odds ratio, 95% CI): both day of/before surgery 0.95 (0.83–1.10), p = 0 0.51, Day before surgery p = 0.91 (0.81–1.02) 0.10, Day of surgery 0.97 (0.88–1.07), p = 0.56 ICU length of stay (adjusted odds ratio, 95% CI): both day of/before surgery 1.12 (0.87–1.45), p = 0.15, Day before surgery 0.92 (0.75–1.13) 0.44, Day of surgery 1.04 (0.84–1.29), p = 0 0.69. Infection, (adjusted odds ratio 95% CI): both day of/before surgery 1.37 (0.81–2.33), p = 0 0.25, day before surgery 1.56 (0.96–2.52), p = 0.07, day of surgery 1.07 (0.66–1.73), p = 0.78	Steroid dose and regimens not reported. No outcomes on inotrope requirements, low cardiac output syndrome, fluid retention or renal failure.
Elhoff et al., Oct. 2015, Pediatr Crit Care Med, USA (7)	549 neonates underwent a Norwood procedure were included. Groups were compared to determine if outcomes differed between intraoperative steroid recipients (n = 498, 91%) and non-recipients (n = 51, 9%).	Multicentre retrospective study (Level 3)	Discharged alive, mechanical ventilation, ICU and hospital length of stay, renal failure, extracorporeal membrane oxygenation (ECMO), psychomotor development index, mental development index.	Steroids vs. non steroid recipients (univariate): hospital survival (94% vs 83%, p = 0.03), ICU stays (16 days; IQR = 12–33 vs. 14 days, IQR = 9–28; p = 0.04) and hospital stays (29 days; interquartile range, 21–50 vs 23 days; interquartile range, 15–40; p = 0.01), ECMO use: 54 (11%) vs. 2 (4%),p = 0.15 infectious complication (197 (40%) vs. 23 (45%), p = 0.44), renal failure (44 (9%) vs. 2 (4%), p = 0.30, psychomotor development index (72 (54–92) vs. 75 (62–94), p = 0.14, mental development Index (92 (77–101) vs. 95 (84–103), p = 0.22.	In multivariate analysis, hospital survival trended toward favouring the non-steroid group with an odds ratio of 3.52 (95% CI, 0.98–12.64; p = 0.054) and lengths of stay associations were no longer significant. Steroid dose and regimens not reported.
Dreher et al. Sept 2015, J Extra Corpor Technol (11)	222 children (55 neonates) undergoing heart surgery without methylprednisolone in CPB prime were compared to 303 children (58 neonates) with methylprednisolone (30 mg/kg up to a maximum dose of 500 mg).	Single centre retrospective study (Level 3)	Death prior to discharge, wound infection, intubation reintubation, respiratory failure requiring tracheostomy, length of stay (days), renal failure, postoperative mechanical circulatory support support.	Neonate outcome data for steroid vs. no steroids: death prior to discharge; 4 (7.27%) vs 6 (10.53%), p-value not significant (ns); any wound infection: 4 (7.27%) vs. 1 (1.75%), p = ns; intubation time (days): 1.29 (0.11–58.27) vs 1.21 (0.1–11.07), p = ns; reintubation: 13 (24.07%) vs. 7 (12.96%), p = ns; respiratory failure requiring tracheostomy: 0 (0.00%) vs 0 (0.00%), p = ns; length of stay (days): 19 (5–179) vs. 15 (3–71), p = ns; renal failure requiring dialysis: 0 (0.00%) vs. 0 (0.00%), p = ns; postoperative mechanical circulatory support: 5 (9.09%) vs. 6 (10.53%), p = ns.	
Ando et al., Ann Thorac Surg, November 2005, Japan (12)	20 neonates undergoing biventricular repair were enrolled; 10 patients received hydrocortisone infusion (0.18 mg kg^−1^ h^−1^ for 3 days, 0.09 mg kg^−1^ h^−1^ for 2 days, and 0.045 mg kg^−1^ h^−1^ for 2 days) after discontinuation of cardiopulmonary by-pass the other 10 received placebo. Patient assignment was manipulated so that inter-anatomic variations were matched between the two groups	Non-randomized controlled trial (Level 3)	Left ventricular shortening fraction, serum lactate level, inotropic score, net fluid balance, A-a gradient, duration of mechanical ventilation, creatinine, urine output, radiologic soft tissue index, blood glucose, renal failure.	Placebo vs. hydrocortisone group: left ventricular shortening fraction (%): 19.0 ± 17.5 vs. 23.4 ± 13.2, p = 0.0203; inotropic score: 9.1 ± 3.0 vs. 7.6 ± 3.4, p = 0.043), serum lactate: 3.3 ± 1.0 vs. 2.3 ± 1.1,p = 0.049; net positive fluid balance (ml/kg): 15.4 ± 28.9 vs. 13.7 ± 24.9, p = 0.027, radiologic soft tissue index: 2.0 ± 0.6 vs. 1.6 ± 0.4, p = 0.065, alveolar–arterial oxygen tension difference (mmHg): 243.1 ± 72.0 vs. 361.2 ± 129.3; duration of mechanical ventilation (83.5 ± 42.1 versus 138.2 ± 89.7 h; p = 0.098), blood glucose: 108.7 ± 18.6 vs. 133.3 ± 45.9,p = 0.152, urine output (mL/kg): 63.1 ± 27.7 vs. 94.8 ± 41.6, p = 0.063.	Both groups received methylprednisolone, 30 mg/kg, at induction. No deaths in both groups.
